# Atresia of the Right Posterior Naris in a Mare

**Published:** 1897-05

**Authors:** W. L. Williams

**Affiliations:** Professor of Surgery, New York State Veterinary College


### ATRESIA OF THE RIGHT POSTERIOR NARIS IN A MARE.

By W. L. Williams, V.S.,
PROFESSOR OF SURGERY, NEW YORK STATE VETERINARY COLLEGE.

The subject, a small brown mare, aged four years, used for delivery by a retail butcher, had been recently purchased without a reliable history as to the date of the advent of respiratory difficulty, but it was said that she had suffered from an increasing dyspnœa for about one year.

As this is the date at which the animal would ordinarily be first put to work, in all probability the difficulty in breathing was merely not noticed until brought out prominently by labor.

Presented at the free clinic, October 9, 1896, it was observed that rapid driving caused severe dyspnœa and roaring, while at rest the respiratory sounds were normal, but the ingress and egress of air were confined wholly to the left nostril, the right being functionless.

Inspection revealed no facial deformity, no definite dulness nor want of resonance over any of the facial sinuses, no nasal discharge or odor, no cough, and no abnormality upon manual exploration of the mouth and pharynx.

The symptoms observed, in conjunction with the history of a gradually developing dyspnœa of a year's duration, led us to believe that we had to deal with a neoplasm encroaching upon the right nasal conduit.

On the following day the animal was cast and two exploratory openings made—one into the inferior maxillary sinus, near the fang of the fourth molar; the other against the median line of the face, on a level with the lower margin of orbits. No abnormality could be discovered from either, except that the turbinated bones, normal in structure, approached more nearly to the septum nasi than usual, until an attempt was made to pass a sound from the second opening through the posterior naris, which was found impossible.

After a prolonged effort and the use of as great force as seemed prudent the sound suddenly passed through into the pharynx, and upon its withdrawal a small quantity of air passed out through the right nostril, and blood passed from the nostrils into the pharynx, leading us to the erroneous conclusion that we had encountered a neoplasm and had passed the sound alongside between it and the bony walls of the posterior naris.

We were unable to learn the form, character, size, or attachments of the obstruction; concluded that a successful operation was impossible, and abandoned further attempts at surgical interference.

It was noticed that the patient now forced some air through the right nostril, and continued to do so; but its significance was not understood, and believing restoration of the animal to usefulness impossible, she was destroyed on November 26th and an autopsy made.

No neoplasm was found, but across the right posterior naris, continuous with the nasal mucosa on the outer and the pharyngeal mucosa on the inner side, was a thin, hymen-like membrane, stretching like a drum-head between the palatine, ethmoid, and vomer bones, completely occluding the opening except for a small rent in its centre, oval in form, three-fourths by one-half inch in diameter, the result of the accidental opening made with the sound at the time of our exploratory operation. The occluded right posterior naris measured transversely from vomer to palatine bone seven-eighths of an inch, while the left measured one and one-half inches. The tissues were all healthy and showed no evidence of pre-existing disease of any kind, indicating clearly that the abnormality was congenital.

*The cause* of the deformity must be referred to early embryonic life after the endoderm of the ovum had, by infolding, produced the primitive intestine, ending anteriorly in the pharynx, in front of which the olfactory pits develop, but are for a time separated from the pharynx by a thin septum, which, becoming obliterated, brings about the opening known as the posterior naris.

In this individual the septum had become obliterated on the left side, while on the right the development had become arrested, the septum, as a result, persisting.

*The bibliography* relating to this form of arrested development is exceedingly limited.

Through the aid of my colleague, Dr. Law, I have been enabled to find the record of one similar case by Prof. Gamgee (*Our Domestic Animals in Health and Disease,* p. 622), who relates an instance observed by Hering in 1842, in which a filly, two and one-half years old, was presented to Prof. Hering for advice regarding a severe dyspnœa and roaring, which had been observed for a year. No evidence of tumor or other neoplasm or pathological condition could be found to explain the roaring, except that it was found that the right nostril was impervious to air and that a flexible sound could not be passed into the pharynx through the right nasal passage. Failing, like me, to make a diagnosis, the filly was destroyed, and, as in our own case, the autopsy revealed a thin septum stretched across and completely closing the right posterior naris, and, in full accord with our views in this case, he considered the cause an arrest in development in the early embryonic stage, by which the septum, at that time normal, failed to undergo that obliteration which should naturally follow.

Perhaps the deformity is more common than records would indicate, and it would seem not unlikely that in some cases both septa persist, leading, especially in foals, to early death, owing to the difficulty it has in breathing through the mouth; hence it would seem well for veterinarians to have in mind the possibility of the occurrence of this peculiar form of arrested development, its diagnosis and treatment.

*The diagnosis* offers no great difficulty to the veterinarian cognizant of the occurrence of such an abnormality. We observe:

*a.* Dyspnœa and roaring.

*b.* Imperviousness of the affected passage to air.

*c.* The absence of any neoplasm or tumors in the nasal passages or sinuses, or of dental or other diseases leading to suppuration or other changes capable of interfering with respiration.

*d.* The nasal passage and nostril free; though, perhaps owing to non-use, the turbinate bones are nearer to the nasal septum than ordinarily observed.

*e.* The posterior naris closed, as shown by the impossibility of passing a sound into the pharynx, but permitting the sound to pass over the naris until the ethmoid bone is reached.

*f.* The Polansky-Schindelka rhino-laryngoscope would enable one to observe the actual condition of the deformed part.

The Gunther Eustachian catheter should prove an excellent sounding instrument, or in its absence an effective sound of similar form could be improvised—that is, a rod about one-fourth of an inch in diameter, with a slight curve anteriorly, commencing about two inches from the anterior end. With this sound measure the distance from the superior angle of the nostril to the lachrymal angle of the orbit, which will about equal the distance from the inner border of the nostril to the centre of the posterior naris.

Passing the sound along the floor of the nasal chamber, it will be found that when it reaches the point indicated by the measurement, instead of passing *downward* into the pharynx, it glides *upward* for a distance of two or three inches and stops against the ethmoid bone. A common flexible horse-catheter would answer the purpose well, but perhaps not so well as the metallic sound.

*The treatment* is exceedingly simple, and consists merely in pushing the curved end of the metallic sound through the membranous partition, and then enlarging the opening, or an opening may be made alongside of the septum nasi, just below the frontal sinuses, and an ordinary pair of curved forceps of sufficient length passed downward between the septum or vomer, striking the persistent membrane almost at right angles and rendering its rupture and laceration to any degree desired readily accomplished. The removal of the lacerated portions would be quite unnecessary.

---

A satin-lined burial-casket covered the remains of a Long Branch dog, whose master thus attested to the fine qualities the animal exhibited.

